# Diaphragmatic dysfunction and its association with postoperative respiratory support in patients with acute secondary peritonitis: A retrospective cohort study

**DOI:** 10.1097/MD.0000000000051410

**Published:** 2026-10-30

**Authors:** Quanrui Feng, Wenxing Zhang, Wei Dai

**Affiliations:** a Intensive Care Unit, Traditional Chinese and Western Medicine Hospital of Wuhan, Tongji Medical College, Huazhong University of Science and Technology, Wuhan, Hubei Province, People’s Republic of China.

**Keywords:** Acute secondary peritonitis, Diaphragm function, Diaphragm ultrasound, Extubation success, Mechanical ventilation

## Abstract

This study aimed to investigate postoperative diaphragmatic function in patients with acute secondary peritonitis and evaluate its association with respiratory support requirements, pulmonary complications, and clinical outcomes. A total of 100 patients with acute secondary peritonitis who underwent emergency surgical intervention and were subsequently admitted to the ICU between January 2022 and February 2024 were retrospectively enrolled. Based on postoperative 24 to 48 hours diaphragm ultrasonography, patients were categorized into preserved diaphragmatic function (n = 42) and impaired function groups (n = 58). Demographics, inflammatory biomarkers, severity scores (APACHE II, SOFA), TFdi, Ddi, ventilation modality and duration, extubation outcome, pulmonary complications, ICU/hospital length of stay, and 28-day mortality were collected. Intergroup comparisons were performed using *t*-test, χ^2^ test, or Mann–Whitney *U* test. Pearson correlation was applied to evaluate the relationship between diaphragmatic function and inflammatory markers/severity scores, and multivariate logistic regression was used to identify independent predictors of extubation failure and prolonged mechanical ventilation. Among the 100 enrolled patients, the impaired diaphragmatic function group showed significantly higher levels of PCT, CRP, IL-6 (all *P* < .05), and increased SOFA and APACHE II scores (both *P* < .01) compared to the preserved function group. TFdi demonstrated moderate negative correlations with PCT, IL-6, and SOFA scores (r=−0.55, −0.50, −0.49; all *P* < .001), while Ddi was also negatively correlated with inflammation and clinical severity (all *P* < .01). Multivariate analysis indicated that reduced TFdi (OR = 2.82, *P* = .001) and decreased Ddi (OR = 1.97, *P* = .009) were independent predictors of extubation failure, and reduced TFdi markedly increased the risk of mechanical ventilation dependence (OR = 3.21, *P* < .001). Clinically, the impaired diaphragmatic function group had higher rates of pulmonary infection (41.4 vs 19.0%, *P* = .018), ARDS (25.9 vs 9.5%, *P* = .032), and re-intubation (27.6 vs 9.5%, *P* = .014), as well as prolonged ICU and hospital stays (both *P* < .01). The 28-day mortality was also significantly higher (13.8 vs 4.8%, *P* = .048). Diaphragmatic function is a critical determinant of postoperative respiratory outcomes in acute secondary peritonitis. Reduced TFdi and Ddi are strongly associated with failed extubation, prolonged mechanical ventilation, a heightened inflammatory response, and increased pulmonary complications. Early bedside ultrasonographic assessment of diaphragmatic performance may assist in identifying high-risk patients, tailoring respiratory management strategies, and improving postoperative prognosis.

## 1. Introduction

Acute secondary peritonitis is one of the most common yet life-threatening abdominal emergencies in surgical practice. It is frequently caused by gastrointestinal perforation, anastomotic leakage, or appendiceal rupture and is characterized by abrupt onset, rapid progression, intense inflammatory response, and a high risk of infection and sepsis.^[1,2]^ Despite advances in surgical techniques, infection control, and early resuscitation strategies, the mortality rate of these patients remains as high as 20 to 30%, and even higher in severely ill cases.^[3,4]^ Therefore, improving perioperative management and postoperative recovery remains an urgent clinical challenge.

The diaphragm, as the principal muscle of respiration, plays a pivotal role in maintaining spontaneous breathing, airway clearance, and adequate ventilation.^[5]^ However, critically ill patients often develop diaphragmatic dysfunction due to systemic inflammation, surgical stress, sedative and anesthetic effects, and prolonged mechanical ventilation. This dysfunction manifests as reduced muscle thickness, decreased contractile capacity, and respiratory muscle fatigue, potentially leading to hypoventilation and extubation failure.^[6,7]^ Emerging evidence shows that diaphragmatic impairment is significantly associated with prolonged mechanical ventilation, increased pulmonary infection, extended ICU length of stay, and poor outcomes.^[8,9]^ Nevertheless, research specifically addressing postoperative diaphragmatic alterations in acute secondary peritonitis remains scarce. Furthermore, the relationship between diaphragmatic dysfunction and postoperative inflammation, illness severity, and extubation outcomes has not been fully elucidated. Clinical decision-making in this population, therefore, lacks strong evidence support.

In recent years, bedside ultrasound has become an increasingly valuable modality for respiratory muscle assessment due to its noninvasive, real-time, and repeatable nature. It has shown potential in evaluating respiratory reserve, predicting extubation readiness, and optimizing ventilatory management among critically ill patients.^[10,11]^ However, whether perioperative diaphragmatic ultrasonography can effectively predict extubation outcomes, reduce ventilator-associated complications, and improve clinical prognosis specifically in acute secondary peritonitis still requires further investigation.

Based on these knowledge gaps, we conducted a retrospective study involving 100 ventilated patients with acute secondary peritonitis. Diaphragm thickness, thickening fraction, and right-sided diaphragmatic mobility were measured using bedside ultrasonography. We aimed to determine the association between diaphragmatic dysfunction and inflammatory burden, illness severity, extubation success, ventilatory dependence, postoperative complications, and clinical outcomes. The findings of this study are expected to provide objective physiological indicators to guide respiratory management and extubation decision-making and to support evidence-based optimization of perioperative treatment strategies in this population.

## 2. Materials and methods

### 2.1. Study design and patient population

This study was approved by the Ethics Committee of Traditional Chinese and Western Medicine Hospital of Wuhan. This was a single-center retrospective cohort study conducted in the Department of Critical Care Medicine of our hospital. Patients who underwent emergency surgical intervention for acute secondary peritonitis and were subsequently admitted to the intensive care unit (ICU) between January 2022 and February 2024 were screened for eligibility. Clinical data, ultrasound measurements, laboratory parameters, ventilatory information, and outcome variables were extracted from the electronic medical record system and the institutional critical care database.

The study complied with the ethical principles of the Declaration of Helsinki and was approved by the institutional ethics committee. Given that this was a retrospective observational analysis without additional patient intervention, the requirement for informed consent was waived.

## 3. Inclusion and exclusion criteria

### 3.1. Inclusion criteria

Patients were eligible if they met all of the following conditions: Age ≥ 18 years; Diagnosed with acute secondary peritonitis confirmed intraoperatively (e.g., gastrointestinal perforation, ruptured appendix, gangrenous cholecystitis, postoperative intra-abdominal infection); Required postoperative ICU admission with respiratory monitoring and had available diaphragm ultrasound measurements within 48 hours after surgery; Complete clinical, ventilatory, and laboratory data records.

### 3.2. Exclusion criteria

Patients were excluded if they had: preexisting neuromuscular disorders affecting diaphragmatic activity (e.g., myasthenia gravis, amyotrophic lateral sclerosis); Chronic respiratory failure or prolonged mechanical ventilation prior to admission; Major thoracic trauma, diaphragmatic rupture, or prior thoracoabdominal surgery interfering with sonographic assessment; End-stage liver or renal failure, terminal malignancy, pregnancy, or incomplete medical records.

## 4. Grouping strategy

A total of 100 patients meeting the criteria were enrolled. Diaphragmatic function was assessed using ultrasonography at 48 hours postoperatively, with measurements including diaphragmatic thickness (Tdi), diaphragm thickening fraction (TFdi), and diaphragmatic excursion (Ddi). Based on previous evidence regarding respiratory muscle fatigue thresholds and combined with pilot observations, TFdi ≥ 20% and Ddi ≥ 10 mm were considered reflective of preserved diaphragmatic contractility. Patients meeting both thresholds were classified into the preserved-diaphragm-function group (n = 42), while those with either TFdi < 20% or Ddi < 10 mm were assigned to the impaired-diaphragm-function group (n = 58). TFdi was used as an index of inspiratory muscle recruitment, whereas Ddi represented mechanical excursion of the diaphragm, and the combination provided a comprehensive evaluation of postoperative respiratory muscle performance. All measurements were performed independently by 2 experienced intensivist sonographers. If inconsistent, a third operator repeated the measurement, and the mean value was recorded. Reliability was validated using intraclass correlation coefficient (ICC) analysis in 20 randomly selected cases, with ICC > 0.80 considered acceptable agreement.

## 5. Data collection

All data were obtained from the electronic medical record system, intraoperative anesthesia records, and ICU critical care database. Two investigators independently extracted and cross-checked the information using a dual-entry validation method to ensure accuracy. The collected dataset included perioperative clinical characteristics, inflammatory biomarkers, severity scores, diaphragm ultrasound indices, postoperative ventilatory parameters, and in-hospital outcomes, detailed as follows:

### 5.1. General clinical and baseline characteristics

Data included age, sex, body mass index (BMI), smoking history, comorbidities (e.g., hypertension, diabetes, chronic kidney disease, COPD), etiology of acute secondary peritonitis (e.g., gastrointestinal perforation, ruptured appendix, gangrenous cholecystitis, anastomotic leakage), surgical approach (laparotomy or laparoscopy), intraoperative blood loss, fluid administration, and antimicrobial strategies.

### 5.2. Laboratory Indicators and Inflammatory Response

Inflammatory biomarkers measured at 24 and 48 hours postoperatively included white blood cell count (WBC), neutrophil percentage, C-reactive protein (CRP), procalcitonin (PCT), and interleukin-6 (IL-6). Arterial blood gas parameters (PaO_2_/FiO_2_ ratio, lactate, pH, PaCO_2_) were also recorded to evaluate postoperative infection severity and tissue oxygenation status.

### 5.3. Disease severity and critical illness scores

Severity of illness was assessed within 24 hours after ICU admission using the Acute Physiology and Chronic Health Evaluation II (APACHE II) score, Sequential Organ Failure Assessment (SOFA) score, clinical respiratory distress indicators, and sepsis-associated diagnostic criteria, reflecting systemic inflammatory burden and organ dysfunction.

### 5.4. Core observational variables

The primary observational indicators were diaphragm ultrasound parameters. Measurements were performed by experienced intensivist sonographers at 24 and 48 hours postoperatively using a high-frequency linear probe. Diaphragm thickness (Tdi) at end-inspiration and end-expiration was recorded to calculate diaphragm thickening fraction (TFdi): (TFdi=(Tdi-end − Tdi-exp)/ Tdi-exp × 100%) . Diaphragmatic excursion (Ddi) was also measured, with 3 consecutive readings averaged for analysis. For suboptimal image quality, repeated assessments were performed to ensure reliability.

No protocolized serial diaphragm ultrasonography beyond the first 48 postoperative hours was available for this retrospective analysis; therefore, the study did not capture the full longitudinal evolution of diaphragmatic function during the subsequent ICU course.

### 5.5. Postoperative respiratory support and clinical outcomes

Collected indicators included ventilatory management (use of invasive ventilation, mode, tidal volume, PEEP level), total duration of mechanical ventilation, requirement for noninvasive ventilation transition, timing and success of first extubation, and re-intubation events. Postoperative complications were recorded, including pneumonia, acute respiratory distress syndrome (ARDS), ICU length of stay, total hospital stay, and in-hospital mortality.

Final endpoint assessments were independently adjudicated by 2 reviewers. Discrepant results were resolved by a third senior investigator to ensure accuracy and consistency.

Because this was a retrospective analysis of routinely collected clinical data, no prespecified formal blinding protocol was implemented. The sonographers could have been aware of contemporaneous clinical information, and the outcome adjudicators were not specifically blinded to diaphragm-function classification. To mitigate potential observer bias, ultrasound measurements were independently performed by 2 experienced intensivist sonographers, with a third operator involved when measurements were inconsistent, and endpoints were independently adjudicated by 2 reviewers with discrepancies resolved by a third senior investigator.

## 6. Study endpoints

Primary and secondary endpoints were defined to comprehensively evaluate the impact of diaphragmatic function on postoperative ventilatory requirements and clinical outcomes.

### 6.1. Primary endpoints

Mechanical ventilation dependence: defined as a duration of invasive mechanical ventilation ≥72 hours postoperatively or the need for re-intubation following initial extubation failure. This indicator reflects the direct influence of diaphragmatic dysfunction on ventilatory support demand.

First-attempt extubation success rate: defined as the ability to maintain spontaneous breathing without re-intubation for at least 48 hours after the initial extubation. Patients requiring re-intubation were categorized as extubation failure, allowing assessment of diaphragm function in predicting autonomous respiratory recovery.

### 6.2. Secondary endpoints

Postoperative pulmonary complications: including hospital-acquired pneumonia, acute respiratory distress syndrome (ARDS), and atelectasis, confirmed via clinical diagnosis and radiographic imaging.

ICU and total hospital length of stay: recorded as the number of ICU days and total hospitalization days, reflecting recovery trajectory and resource utilization.

In-hospital and 28-day mortality: defined as death occurring during hospitalization or within 28 days after admission, serving as a key measure of short-term prognosis associated with diaphragm dysfunction.

All endpoint data were extracted from electronic medical records and ICU monitoring systems by 2 independent investigators and cross-validated. Discrepancies were resolved by a third senior reviewer to ensure accuracy and reliability.

## 7. Statistical analysis

All statistical analyses were performed using SPSS version 26.0. Normality of continuous variables was tested prior to analysis. Normally distributed data were presented as mean ± standard deviation (x̄ ± s) and compared using the independent-sample t-test; non-normally distributed data were expressed as median and interquartile range [M (Q1, Q3)] and compared using the Mann–Whitney *U* test. Categorical variables were described as counts and percentages [n (%)] and compared using the χ^2^ test or Fisher’s exact test. Correlations between diaphragmatic ultrasound parameters (Tdi, TFdi, Ddi) and inflammatory biomarkers (CRP, PCT, IL-6) as well as severity scores (APACHE II, SOFA) were assessed using Pearson or Spearman correlation analysis, with correlation coefficients (r) and *P*-values reported. Potential predictors of first-attempt extubation success and mechanical ventilation dependence were initially screened using univariate logistic regression. Variables with *P* < .1 were subsequently entered into multivariate logistic regression to identify independent risk factors. Results were expressed as odds ratios (ORs) with 95% confidence intervals (CIs). A two-tailed *P* < .05 was considered statistically significant.

## 8. Results

### 8.1. Baseline characteristics and overall clinical profile of the study population

A total of 100 patients with acute secondary peritonitis were enrolled in this study, including 42 in the preserved diaphragmatic function group and 58 in the impaired function group. There were no significant differences between the 2 groups in terms of sex distribution, BMI, smoking history, or underlying comorbidities (*P* > .05), indicating good comparability at baseline. Patients with diaphragmatic dysfunction were slightly older than those with preserved function (58.6 ± 12.1 vs 53.4 ± 11.6 years, *P* = .037) and exhibited a higher rate of preoperative shock (43.1 vs 21.4%, *P* = .018), suggesting a potential association between diaphragmatic impairment and more severe pathological conditions. Disease severity scores also differed significantly between groups: APACHE II and SOFA scores were higher in the dysfunction group (APACHE II: 16.0 ± 6.2 vs 13.1 ± 5.3, *P* = .012; SOFA: 6.8 ± 2.6 vs 5.3 ± 2.3, *P* = .009). Regarding inflammatory markers, levels of CRP, PCT, and IL-6 were elevated in the dysfunction group, with CRP and PCT showing statistically significant differences (*P* < .05). WBC count also demonstrated an upward trend. Additionally, PaO_2_/FiO_2_ was lower in patients with diaphragmatic dysfunction (255.8 ± 69.4 vs 284.6 ± 58.5 mm Hg, *P* = .028), indicating compromised oxygenation capacity. Overall, patients with impaired diaphragmatic function presented with more pronounced systemic inflammatory activation, greater physiological burden, and overall more severe clinical status (Table 1).

**Table 1:** Baseline characteristics and perioperative clinical data between the two groups.

Variables	Total (n = 100)	Normal diaphragm function (n = 42)	Impaired diaphragm function (n = 58)	*P* value
**Demographic data**				
Age (years, mean ± SD)	56.3 ± 12.0	53.4 ± 11.6	58.6 ± 12.1	.037*
Male, n (%)	62 (62.0)	25 (59.5)	37 (63.8)	.731
BMI (kg/m^2^, mean ± SD)	23.7 ± 3.8	23.3 ± 3.6	24.0 ± 3.9	.354
Smoking history, n (%)	29 (29.0)	10 (23.8)	19 (32.8)	.321
**Comorbidities**				
Hypertension, n (%)	36 (36.0)	13 (31.0)	23 (39.7)	.365
Diabetes mellitus, n (%)	30 (30.0)	10 (23.8)	20 (34.5)	.249
COPD, n (%)	9 (9.0)	3 (7.1)	6 (10.3)	.739
**Disease severity indicators**				
Preoperative shock, n (%)	32 (32.0)	9 (21.4)	25 (43.1)	.018*
APACHE II score (mean ± SD)	14.8 ± 5.9	13.1 ± 5.3	16.0 ± 6.2	.012*
SOFA score (mean ± SD)	6.2 ± 2.5	5.3 ± 2.3	6.8 ± 2.6	.009*
**Inflammatory biomarkers**				
CRP (mg/L, median [IQR])	149 [110–192]	134 [95–172]	161 [122–205]	.033*
PCT (ng/mL, median [IQR])	3.75 [2.02–6.14]	2.68 [1.35–4.45]	4.48 [2.90–6.92]	.012*
IL-6 (pg/mL, mean ± SD)	61.3 ± 28.4	55.2 ± 24.9	65.6 ± 29.8	.074
Respiratory parameters				
PaO_2_/FiO_2_ (mm Hg, mean ± SD)	268.1 ± 65.3	284.6 ± 58.5	255.8 ± 69.4	.028*

* *P* < .05 indicates statistical significance.

### 8.2. Comparison of postoperative diaphragm function parameters between successful and failed extubation/dependent group patients based on the outcome

We further compared early postoperative diaphragmatic function between the successful extubation group and the extubation failure/ventilator-dependent group. Significant differences were observed in diaphragmatic excursion, diaphragmatic thickening fraction (DTF), and phrenic nerve conduction time within 24 hours after surgery. Diaphragmatic excursion was markedly reduced in patients who experienced extubation failure or required prolonged ventilatory support (right side: 6.8 ± 2.1 mm vs 11.2 ± 3.4 mm; left side: 7.1 ± 2.3 mm vs 10.7 ± 3.1 mm; all *P* < .001). Similarly, DTF values were significantly lower (right: 26.5 ± 7.8% vs 42.3 ± 10.5%; left: 27.8 ± 8.3% vs 40.9 ± 9.7%; all *P* < .001). Phrenic nerve conduction time was prolonged in the extubation failure/ventilation-dependent group (16.9 ± 3.9 ms vs 13.2 ± 2.8 ms, *P* < .001), indicating reduced phrenic nerve excitability. The incidence of postoperative diaphragmatic dysfunction (defined as DTF < 30% or excursion < 10 mm) was significantly higher in patients with extubation failure or ventilator dependence (47.1 vs 15.4%, *P* < .001). These findings suggest that postoperative diaphragmatic contractility and dynamic thickening are notably impaired in patients with acute secondary peritonitis, and that reduced diaphragmatic performance is strongly associated with a higher likelihood of extubation failure or continued ventilatory dependence (Table 2).

**Table 2:** Comparison of postoperative diaphragmatic function between the extubation success group and the extubation failure/ventilation dependence group.

Variable	Extubation success (n = 65)	Extubation failure/ventilation dependence (n = 35)	Statistics (t/χ^2^)	*P* value
**Right diaphragmatic excursion (mm**)	11.2 ± 3.4	6.8 ± 2.1	t = 7.42	<.001
**Left diaphragmatic excursion (mm**)	10.7 ± 3.1	7.1 ± 2.3	t = 6.11	<.001
**Right diaphragmatic thickening fraction (%**)	42.3 ± 10.5	26.5 ± 7.8	t = 8.29	<.001
**Left diaphragmatic thickening fraction (%**)	40.9 ± 9.7	27.8 ± 8.3	t = 7.21	<.001
**Diaphragmatic nerve conduction time (ms**)	13.2 ± 2.8	16.9 ± 3.9	t = −4.96	<.001
**Diaphragmatic dysfunction (%**)*	10 (15.4%)	16 (47.1%)	χ^2^ = 11.56	<.001

* Diaphragmatic dysfunction was defined as diaphragmatic excursion < 10 mm or DTF < 30%.

### 8.3.3. Correlation between diaphragmatic dysfunction, inflammatory severity, and disease scoring

To further evaluate the association between diaphragmatic functional status and postoperative inflammatory response and clinical severity, Pearson correlation analysis was performed to assess the relationships of diaphragmatic excursion (Ddi) and diaphragmatic thickening fraction (TFdi) with inflammatory biomarkers (WBC, CRP, PCT, IL-6) and severity scores (SOFA, APACHE II). The results revealed that lower diaphragmatic function – characterized by reduced excursion and thickening – was associated with higher levels of postoperative inflammation and increased illness severity, demonstrating moderate to strong correlation patterns. Specifically, Ddi was significantly negatively correlated with CRP, PCT, and IL-6 (r = −0.41, −0.52, and − 0.48, respectively; all *P* < .01), indicating that restricted diaphragmatic motion was linked to a more pronounced postoperative inflammatory response. TFdi also exhibited negative correlations with inflammatory markers, with the strongest associations observed for PCT and IL-6 (r = −0.55 and − 0.50, both *P* < .001). Meanwhile, diaphragmatic function was significantly correlated with disease severity. Ddi was inversely associated with both SOFA and APACHE II scores (r = −0.46 and − 0.43, both *P* < .01), while TFdi showed an even stronger correlation with SOFA (r = −0.49, *P* < .001), suggesting a close link between diaphragmatic impairment and the risk of multi-organ dysfunction. Overall, these findings demonstrate an inverse relationship between postoperative diaphragmatic function and systemic inflammatory burden. Patients with poorer diaphragmatic performance exhibited greater infectious and inflammatory activation, more severe physiological insult, and more critical ICU progression. Diaphragm ultrasound may therefore serve as a valuable indicator for assessing postoperative inflammatory intensity and disease severity (Table 3).

**Table 3:** Correlation analysis between diaphragm function and inflammatory markers/ severity scores.

Variables	Correlation coefficient (r)	*P* value
**Ddi vs WBC count**	−0.28	.012
**Ddi vs CRP**	−0.41	<.001
**Ddi vs PCT**	−0.52	<.001
**Ddi vs IL-6**	−0.48	<.001
**TFdi vs WBC count**	−0.31	.006
**TFdi vs CRP**	−0.44	<.001
**TFdi vs PCT**	−0.55	<.001
**TFdi vs IL-6**	−0.50	<.001
**Ddi vs SOFA score**	−0.46	<.001
**Ddi vs APACHE II score**	−0.43	<.001
**TFdi vs SOFA score**	−0.49	<.001
**TFdi vs APACHE II score**	−0.40	<.001

APACHE II = Acute Physiology and Chronic Health Evaluation II, CRP = C-reactive protein, Ddi = diaphragm displacement, IL-6 = interleukin-6, PCT = procalcitonin, SOFA = Sequential Organ Failure Assessment, TFdi = diaphragm thickening fraction, WBC = white blood cell count.

### 8.4. Multivariate analysis of predictors of extubation success and risk of ventilator dependence

To identify independent predictors of extubation outcome and ventilator dependence, multivariate logistic regression was performed using first-attempt extubation failure and mechanical ventilation dependence as outcome variables. Covariates included age, BMI, intraoperative blood loss, APACHE II score, SOFA score, PCT level, diaphragmatic thickening fraction (TFdi), and diaphragmatic excursion (Ddi). The analysis demonstrated that diaphragmatic function parameters were the strongest independent determinants of extubation outcome. Among them, TFdi was the most powerful predictor of extubation failure (OR = 2.82, 95% CI: 1.54–5.15, *P* = .001), while reduced Ddi was also independently associated with increased failure risk (OR = 1.97, 95% CI: 1.18–3.30, *P* = .009). In terms of systemic inflammation, elevated PCT levels significantly increased the likelihood of extubation failure (OR = 1.56, 95% CI: 1.11–2.20, *P* = .011), suggesting that infection and inflammatory activation may aggravate diaphragmatic fatigue and hinder respiratory recovery. Additionally, higher SOFA scores were independently associated with extubation failure (OR = 1.44, 95% CI: 1.07–1.93, *P* = .017). Regarding mechanical ventilation dependence, reduced TFdi was strongly correlated with prolonged ventilatory support (OR = 3.21, 95% CI: 1.78–5.79, *P* < .001), and diminished Ddi likewise increased the risk of ventilator dependence (OR = 2.35, 95% CI: 1.29–4.28, *P* = .005). Higher APACHE II scores were also associated with increased likelihood of ventilation dependence (OR = 1.32, 95% CI: 1.03–1.69, *P* = .028). Among inflammatory markers, the association of PCT and IL-6 was most consistent, though only PCT remained in the final predictive model for ventilator dependence. Collectively, these findings indicate that decreased diaphragmatic function – as reflected by reductions in TFdi and Ddi – significantly increases the risk of extubation failure and mechanical ventilation dependence. These associations persist independent of baseline patient characteristics, inflammatory status, and disease severity scores, highlighting diaphragmatic dysfunction as a crucial determinant of postoperative respiratory support requirements (Table 4).

**Table 4:** Multivariate logistic regression analysis of factors associated with extubation failure and mechanical ventilation dependency.

Variables	OR for Extubation Failure	95% CI	*P* value	OR for Ventilation Dependency	95% CI	*P* value
**Reduced TFdi**	2.82	1.54–5.15	.001	3.21	1.78–5.79	<.001
**Reduced Ddi**	1.97	1.18–3.30	.009	2.35	1.29–4.28	.005
**Elevated PCT**	1.56	1.11–2.20	.011	1.48	1.05–2.01	.023
**Higher SOFA score**	1.44	1.07–1.93	.017	1.38	0.99–1.89	.056
**Higher APACHE II score**	1.29	0.96–1.72	.081	1.32	1.03–1.69	.028
**Age**	1.03	0.99–1.07	.112	1.02	0.98–1.06	.184
**BMI**	1.08	0.94–1.23	.243	1.05	0.92–1.20	.391

APACHE II = Acute Physiology and Chronic Health Evaluation II, BMI = body mass index, Ddi = diaphragm displacement, PCT = procalcitonin, SOFA = Sequential Organ Failure Assessment, TFdi = diaphragm thickening fraction.

### 8.5. Association between diaphragmatic function and postoperative complications and clinical outcomes

To further evaluate the impact of postoperative diaphragmatic function on recovery and clinical prognosis, we compared pulmonary complications, ICU and total hospital length of stay, and mortality between patients with preserved vs impaired diaphragmatic function. The incidence of postoperative complications was significantly higher in the impaired-function group (n = 58) compared with the preserved-function group (n = 42). Specifically, rates of pulmonary infection were 41.4 vs 19.0% (*P* = .018), and ARDS occurred more frequently in patients with diaphragmatic impairment (25.9 vs 9.5%, *P* = .032). The impaired group also had markedly higher reintubation and extubation failure rates (27.6 vs 9.5%, *P* = .014) and required noninvasive ventilation transition more often (36.2 vs 16.7%, *P* = .021). In terms of clinical outcomes, ICU stay was prolonged in patients with reduced diaphragmatic function (median 6.5 vs 4.0 days, *P* = .004), and total length of hospitalization was also significantly longer (14.0 vs 10.5 days, *P* = .009). The 28-day mortality rate was higher in the impaired group compared with the preserved group (13.8 vs 4.8%, *P* = .048), suggesting that diaphragmatic dysfunction not only hinders respiratory recovery but may also be linked to systemic organ deterioration and poorer short-term prognosis. Taken together, postoperative diaphragmatic dysfunction is associated with an increased risk of pulmonary infection and ARDS, greater respiratory support requirements, prolonged ICU monitoring, higher reintubation rates, and reduced short-term survival. These findings indicate that diaphragmatic function may serve as a valuable prognostic indicator for clinical outcome, aiding in the early identification of high-risk patients and guiding optimization of respiratory support strategies (Table 5).

**Table 5:** Comparison of postoperative complications and clinical outcomes between patients with preserved vs impaired diaphragm function.

Outcomes	Preserved diaphragm function (n = 42)	Impaired diaphragm function (n = 58)	*P* value
**Pulmonary infection, %**	19.0	41.4	.018
**ARDS, %**	9.5	25.9	.032
**Extubation failure/ reintubation, %**	9.5	27.6	.014
**Need for noninvasive ventilation transition, %**	16.7	36.2	.021
**Duration of mechanical ventilation (days, IQR**)	2.8 (1.6–4.3)	4.9 (3.1–7.4)	.003
**ICU length of stay (days, IQR**)	4.0 (3.0–6.0)	6.5 (4.5–9.0)	.004
**Total hospital stay (days, IQR**)	10.5 (8.0–14.0)	14.0 (10.0–19.5)	.009
**28-day mortality, %**	4.8	13.8	.048

ARDS = acute respiratory distress syndrome, ICU = intensive care unit, IQR = interquartile range.

## 9. Discussion

This study investigated the association between postoperative diaphragmatic dysfunction and extubation outcomes, ventilator dependence, and clinical prognosis in patients with acute secondary peritonitis. Our results indicate that diaphragmatic impairment is closely linked to a heightened postoperative inflammatory response and greater illness severity, and moreover represents the strongest independent predictor of extubation failure and prolonged mechanical ventilation. These findings highlight the substantial clinical value of diaphragmatic ultrasound monitoring in the respiratory management of critically ill surgical patients, providing objective support for extubation timing and ventilator weaning strategies.^[12,13]^

To begin with, patients with impaired diaphragmatic function demonstrated higher APACHE II and SOFA scores, together with a more pronounced inflammatory response, evidenced by significantly elevated CRP and PCT levels. This suggests a consistent relationship between diaphragm dysfunction, systemic inflammatory activation, and disease severity following surgery.^[12,13]^ Correlation analysis further confirmed that diaphragmatic excursion (Ddi) was negatively associated with CRP, PCT, and IL-6, while diaphragm thickening fraction (TFdi) showed moderate-to-strong inverse correlations with inflammatory burden. These results imply that greater diaphragmatic impairment corresponds to more intense infection and immune activation, potentially driven by inflammation-mediated myofiber injury, mitochondrial dysfunction, and impaired neuromuscular transmission, ultimately leading to diaphragmatic fatigue and reduced contractility.^[14,15]^ This observation aligns with the previously proposed “inflammation–diaphragm dysfunction loop,” underscoring the need for proactive respiratory muscle surveillance and intervention in critical care.^[16]^

More importantly, multivariate analysis confirmed that diaphragmatic parameters were the strongest determinants of extubation outcome. TFdi emerged as the most powerful predictor (OR = 2.82, 95% CI 1.54–5.15), with Ddi also independently associated with extubation failure (OR = 1.97, 95% CI 1.18–3.30). Reduced TFdi was likewise strongly linked to ventilator dependence (OR = 3.21, *P* < .001), indicating that insufficient diaphragmatic contractility not only impairs weaning success but also prolongs the need for respiratory support. Notably, the predictive value of diaphragmatic measures remained robust even after adjusting for systemic condition and inflammatory levels, demonstrating their independent and direct clinical relevance.^[17,18]^ Based on these findings, postoperative patients with TFdi < 30% or diaphragmatic excursion < 10 mm should be regarded as high-risk for extubation failure and may benefit from delayed extubation, stepwise transition to noninvasive ventilation, or early respiratory muscle training to reduce complications.

Additionally, the impaired-function group exhibited markedly higher incidences of pulmonary infection and ARDS, as well as increased reintubation rates and more frequent noninvasive ventilation use. ICU stay and overall hospitalization were significantly prolonged, and 28-day mortality was markedly higher (13.8 vs 4.8%). These results suggest that diaphragmatic dysfunction not only predicts extubation outcomes, but is also closely associated with short-term survival, likely through its effects on alveolar recruitment, cough clearance, and respiratory dynamics, thereby predisposing patients to infection, atelectasis, and respiratory failure.^[19,20]^ Thus, diaphragm function serves not merely as a respiratory parameter, but as a physiologic window reflecting systemic inflammation, organ dysfunction, and overall recovery quality.

Several limitations should be acknowledged. First, this was a single-center retrospective cohort study with a relatively small sample, and the use of existing clinical records introduces inherent risks of selection bias and information/recording bias. nonrandom clinical decisions, including the choice of surgical approach, source-control strategy, postoperative ICU management, and respiratory-support strategy, may have influenced both diaphragmatic function and clinical outcomes; therefore, the observed associations should not be interpreted as causal. Second, residual confounding cannot be excluded. Although perioperative variables such as intraoperative fluid administration were collected, potentially important factors were not comprehensively incorporated into the adjusted analyses, including anesthetic technique, the dose and duration of neuromuscular blocking agents, sedative exposure, and detailed fluid-management strategies. These factors may independently affect diaphragmatic contractility and weaning outcomes. Third, diaphragm ultrasonography was confined to the early postoperative period (24 and 48 hours), with no protocolized serial assessment beyond 48 hours. Diaphragmatic function may deteriorate or recover during the subsequent course of sepsis, mechanical ventilation, mobilization, and treatment; thus, early measurements may underestimate or overestimate its longer-term association with respiratory outcomes. Fourth, no prespecified formal blinding protocol was implemented for sonographic assessment or endpoint adjudication, and measurement or diagnostic/adjudication bias therefore cannot be completely excluded despite independent duplicate assessment and discrepancy resolution procedures. Finally, the sample size and number of outcome events may limit model stability and generalizability, and external validation was not performed. Future multicenter prospective studies should incorporate standardized perioperative management, repeated longitudinal diaphragm ultrasound, and broader adjustment for anesthesia-, neuromuscular blockade-, ventilation-, and fluid-related confounders.

In conclusion, this study confirms that diaphragmatic dysfunction plays a pivotal role in postoperative respiratory failure and weaning difficulty. Given its noninvasive, real-time, and repeatable advantages, diaphragmatic ultrasound should be incorporated into routine evaluation for critically ill surgical patients to identify high-risk extubation candidates, guide respiratory muscle rehabilitation, optimize weaning strategies, and ultimately improve clinical outcomes. Future multicenter prospective trials are warranted to establish validated predictive thresholds for TFdi and Ddi and to develop standardized diaphragm-based extubation assessment protocols, with the aim of enhancing recovery and survival in critically ill populations.

## Author contributions

**Conceptualization:** Quanrui Feng, Wenxing Zhang, Wei Dai.

**Data curation:** Quanrui Feng, Wenxing Zhang, Wei Dai.

**Formal analysis:** Quanrui Feng, Wenxing Zhang, Wei Dai.

**Funding acquisition:** Quanrui Feng, Wenxing Zhang, Wei Dai.

**Investigation:** Wei Dai.

**Writing** – **original draft:** Wei Dai.

**Writing** – **review & editing:** Wei Dai.
